# Complete Genome of *Bacillus subtilis* Myophage CampHawk

**DOI:** 10.1128/genomeA.00984-13

**Published:** 2013-12-19

**Authors:** Morgan P. Ritz, Abbey L. Perl, Jennifer M. Colquhoun, Karthik R. Chamakura, Gabriel F. Kuty Everett

**Affiliations:** Center for Phage Technology, Texas A&M University, College Station, Texas, USAa; Department of Biological Sciences, Lehigh University, Bethlehem, Pennsylvania, USAb

## Abstract

The study of bacteriophages infecting the model organism *Bacillus subtilis* has provided an abundance of general knowledge and a platform for advances in biotechnology. Here, we announce the annotated genome of CampHawk, a *B. subtilis* phage. CampHawk was found to be an SPO1-like phage with similar gene content and arrangement.

## GENOME ANNOUNCEMENT

*Bacillus subtilis* is a Gram-positive, sporulating bacterium that is commonly found in soil and in the gut of healthy humans (1). *B. subtilis* is used as a model organism for research fields such as biofilm production, sporulation, and many others. It is also used in industry for fungicide and antibiotic production (2). The study of bacteriophages infecting *B. subtilis* has contributed greatly to our general knowledge of this and other model organisms (3). Here, we present the complete genome of the *B. subtilis* SPO1-like myophage CampHawk.

CampHawk was isolated from a soil sample collected in Bethlehem, PA. Phage DNA was sequenced using 454 pyrosequencing at the Emory GRA Genome Center (Emory University, Atlanta, GA). Trimmed FLX Titanium reads were assembled to a single contig at 131.4-fold coverage using the Newbler assembler, version 2.5.3 (454 Life Sciences), at default settings. The contig was confirmed to be complete by PCR. Genes were predicted using GeneMarkS (4) and corrected using software tools available on the Center for Phage Technology (CPT) portal (https://cpt.tamu.edu/cpt-software/portal/).

CampHawk has a 132,421-bp unit genome, with an 87.9% coding density and an overall G+C content of 40.18%. EMBOSS Stretcher nucleotide alignment shows the genome of CampHawk to be 84.8% identical to that of SPO1 (5). Processing the raw sequencing data using the Pause method (https://cpt.tamu.edu/cpt-software/releases/pause/) determined that CampHawk has a long terminal repeat of 13,772 bp. The unit genome contains 200 coding sequences and three tRNA genes.

The gene arrangement in the CampHawk genome is comparable to that of the SPO1 genome. Genes annotated include those encoding proteins needed for virion assembly, such as the large terminase, portal protein, prohead protease, scaffolding protein, major head protein, tail sheath protein, tail tube, tape measure, baseplate proteins, tail fiber proteins, and a tailspike protein with a pectin lyase domain. As in SPO1, the annotated tail fiber proteins contain putative pectin lyase domains. The genome encodes proteins used for DNA replication and repair, including helicases (DnaB and UvrD), DNA primase, DNA polymerase, ligase, and various nucleases. Genes for biosynthesis proteins were also found (cytidine deaminase, dUTPase, ribonucleoside-diphosphate reductase alpha and beta, and thymidylate synthase). Like SPO1, CampHawk has an intein (InterPro accession no. IPR006141) disrupting the UvrD helicase that spans Cys292-N576 as determined by conserved intein boundaries (6). CampHawk also has an HNH homing nuclease, sigma factors, and other transcriptional regulators. The lysis cassette includes an endolysin (l-alanyl-d-glutamate peptidase) sandwiched between two holins. The first holin has two transmembrane domains (TMDs) in an N-out C-out topology. The second holin has a single TMD in an N-out C-in topology. Likely, one protein is the holin while the other is an antiholin (7). A small acid-soluble spore protein (SASP) gene was identified as well. In sporulating bacteria, SASPs are nonspecific double-stranded DNA (dsDNA) binding proteins that protect spore DNA under harsh conditions, such as ultraviolet light, hydrogen peroxide exposure, and high temperature (8). Presumably, the phage-encoded SASP protects phage DNA in a similar manner.

### Nucleotide sequence accession number.

The genome sequence of phage CampHawk was contributed as accession no. KF669649 to GenBank.
